# 4D Printing in Biomedical Implants and Functional Healthcare Devices

**DOI:** 10.3390/jfb17040203

**Published:** 2026-04-20

**Authors:** Muhammad Shafiq, Liaqat Zeb

**Affiliations:** 1 School of Medical Sciences, Shandong Xiehe University, Jinan 250109, China; shafiqmuhammad@sdxiehe.edu.cn; 2 Laboratory of Bioresources and Pharmaceutical Chemistry, Department of Chemistry, University of Bergen, 5007 Bergen, Norway

**Keywords:** 4D printing, biomedicals implants, drug delivery systems

## Abstract

Four-dimensional (4D) printing integrates additive manufacturing with stimuli-responsive materials to fabricate biomedical implants and functional healthcare devices that undergo programmed, time-dependent changes in shape or function. Unlike static 3D-printed constructs, 4D-printed systems can respond to clinically relevant stimuli such as temperature, hydration, pH, light (including near-infrared), magnetic fields, or electrical inputs. These triggers drive defined actuation mechanisms, most commonly thermomechanical shape-memory recovery, swelling-induced morphing, and magnetothermal activation. This review synthesizes the principal material platforms used for biomedical 4D printing, including shape-memory polymers and alloys, hydrogels, liquid-crystal elastomers, and responsive composites, and links material choice to device behavior and translational feasibility. Applications are discussed across self-expanding stents, cardiac occluders, tissue-engineered constructs, implantable drug delivery systems, and adaptive wearables. Key translational challenges include sterilization compatibility, manufacturing reproducibility and quality control, safe stimulus delivery, predictable biodegradation and long-term biocompatibility, and regulatory pathway definition.

## 1. Introduction

Additive manufacturing (AM), or three-dimensional (3D) printing, constructs complex, patient-specific architectures by depositing material layer-by-layer from computer-aided design (CAD) files [1,2]. Modalities such as inkjet and binder jetting, extrusion printing, selective laser sintering and stereolithography afford high structural fidelity, reproducibility and post-processing flexibility, enabling porous scaffolds that recapitulate meniscus, bone, ear and nasal geometries and supporting applications that range from organ fabrication and drug delivery to medical phantoms and personalised oncology therapies [3,4]. Yet the resulting constructs are typically static: they neither reshape, self-heal nor remodel in response to external cues, and thus fall short of emulating native tissue dynamics [5,6]. 4D printing addresses this limitation by combining AM with stimuli-responsive materials that endow printed objects with programmed, time-dependent morphogenesis.

**Figure 1:**
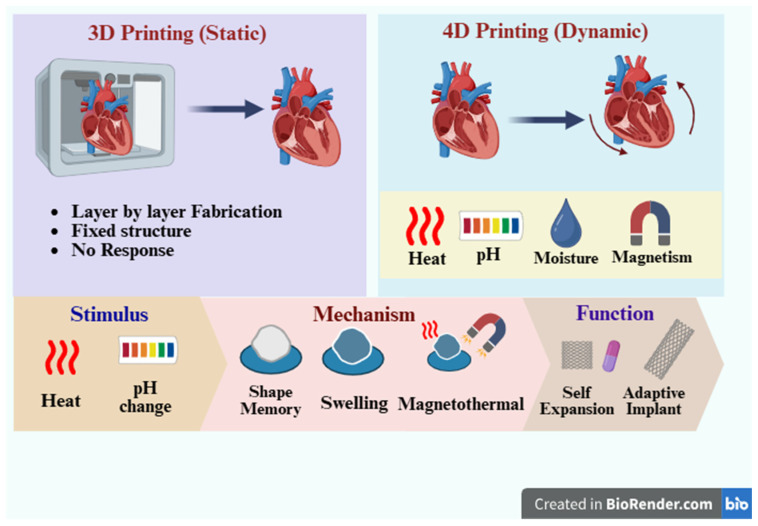
Conceptual comparison between 3D and 4D printing technologies.

Four-dimensional (4D) printing has emerged as a conceptual and technological extension of three-dimensional (3D) printing [7,8]. Rather than representing an entirely new manufacturing technique, 4D printing arises from the integration of additive manufacturing with smart or stimulus-responsive materials. In this framework, a printed object is programmed during fabrication to undergo a controlled, time-dependent transformation in response to predefined environmental triggers [9,10,11]. The “fourth dimension” refers to time, during which the structure changes its shape, mechanical properties, or function after printing [3,12]. The functionality of 4D printing fundamentally relies on stimuli-responsive material systems, including shape-memory polymers (SMPs), hydrogels, liquid crystal elastomers (LCEs), and composite materials incorporating magnetic or photothermal fillers [13,14]. These materials undergo predictable transformations when exposed to specific stimuli, including temperature changes, moisture, pH variation, light, magnetic fields, or electrical signals (Figure 1). Consequently, 4D-printed constructs can achieve self-deployment, reversible morphing, stiffness modulation, and controlled therapeutic release [13,15,16]. To improve conceptual clarity, 4D printing systems can be classified according to the stimulus and response types.

Classification by Stimulus Type

- Thermal stimuli include body temperature activation, external heating, and near-infrared (NIR) photothermal conversion.
- Moisture or hydration stimuli: Water-induced swelling or anisotropic expansion of the material.
- pH-responsive systems: Environmental acidity or alkalinity triggers structural changes.
- Light-responsive systems: Photothermal or photocrosslinking-induced transformation.
- Magnetic stimuli: Alternating magnetic fields that produce magnetothermal effects.
- Electrical or biochemical stimuli: Electric-field-induced actuation or biologically mediated responses [3,17,18].

Classification by Response Type

- Shape morphing: Programmable geometric transformation via shape memory or differential swelling.
- Swelling or volumetric expansion: Hydrogel-based deformation occurs.
- Stiffness modulation: Tunable mechanical properties upon activation.
- Drug release: Stimulus-triggered therapeutic delivery.
- Self-assembly or self-folding: Autonomous structural organization after the fabrication [19,20,21].

This dual classification framework highlights that 4D printing is defined not only by shape change but also by programmable, stimulus-driven functionality embedded at the material level during additive manufacturing [3,17,19]. This capability is particularly relevant in healthcare. Biological systems are inherently dynamic, and implants must function in mechanically active, chemically complex, and temporally evolving environments. By enabling constructs that deploy minimally invasively, conform to anatomical variations, accommodate tissue growth, or release therapeutics in a controlled manner, 4D printing presents a promising platform for next-generation biomedical devices and regenerative strategies [20,22].

In the light of recent progress, we review the advances of 4D printing construct towards health care. We have explored its applications spanning tissue engineering, biomedical devices, cardiac occludes, wearables and drug delivery systems. Further, we have discussed challenges and future development in 4D printing of biomedical constructs.

## 2. Methodological Aspects

In 2026, data were systematically collected from peer-reviewed literature through comprehensive searches in the Web of Science, PubMed, and Scopus databases. The search strategy combined key terms relevant to this review, including “4D printing,” “four-dimensional printing,” “stimuli-responsive materials,” “shape-memory polymer,” “4D printing AND implant,” “4D printing and stent,” “4D printing and drug delivery,” and “4D printing and tissue engineering.” Searches were limited to titles and abstracts, and the time window was restricted to publications from January 2015 to December 2026. Approximately 320 articles were identified, and duplicate records were removed prior to screening.

The review is organized into the following subsections: (a) Definitions and classification framework; (b) Materials for 4D printing; (c) Biomedical implants and functional healthcare devices; (d) Translational considerations and clinical challenges; and (e) Future perspectives. Studies were included if they described additive manufacturing integrated with stimuli-responsive materials and reported defined actuation mechanisms in biomedical contexts. Studies focused solely on conventional 3D printing or non-biomedical applications were excluded. Titles and abstracts were screened for relevance, followed by full-text evaluation to confirm eligibility. Relevant data were extracted and analyzed to provide a structured and evidence-based discussion.

## 3. Materials Used in 4D Printing for Biomedical Applications

**Figure 2:**
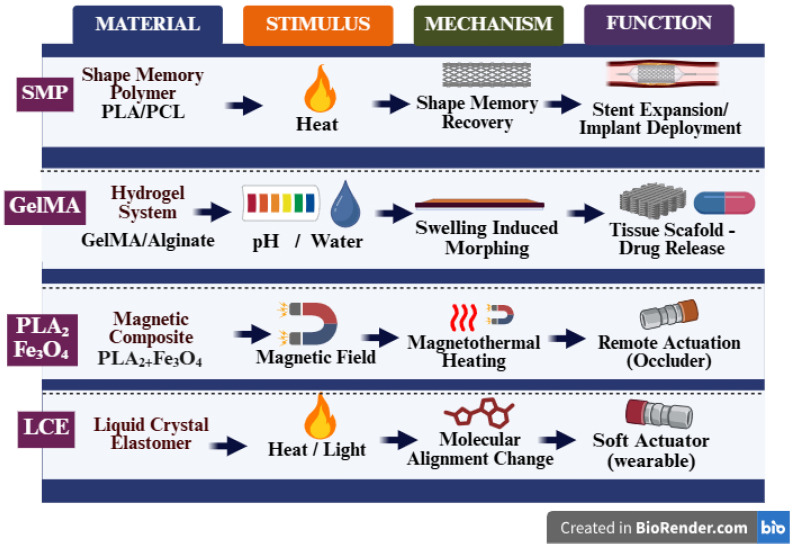
Material stimulus mechanism relationships in 4D-printed biomedical systems.

The performance of 4D-printed biomedical systems is fundamentally determined by the physicochemical properties of the selected stimuli-responsive materials. As summarized in Table 1, 4D-printed systems are classified according to stimulus type, actuation mechanism, reversibility of transformation, and clinical maturity level. Therefore, selection directly influences activation pathway, deformation magnitude, response kinetics, and translational feasibility. In biomedical contexts, material systems must satisfy additional requirements beyond actuation capability. These include cytocompatibility, stability in physiological fluids, sterilization tolerance, mechanical integrity during transformation, and safe activation thresholds. For biodegradable systems, degradation rate must align with tissue healing or therapeutic timelines [23,24]. The major material classes currently employed in 4D biomedical printing are discussed below (Figure 2).

**Table 1:** Classification Framework for 4D-Printed Biomedical Systems.

Category	Subclassification	Description
Stimulus Type	Thermal	Activation near physiological temperature or external heating
	Magnetic	Magnetothermal activation via alternating magnetic fields
	Near-infrared (NIR)	Photothermal conversion using embedded agents
	pH-responsive	Structural changes triggered by environmental pH
	Hydration/swelling	Water-induced expansion in hydrophilic materials
	Electric field	Deformation induced by electrical stimulation
Reversibility	Reversible systems	Multiple cycles of actuation without permanent change
	Irreversible systems	One-time deployment or permanent transformation
Actuation Mechanism	Shape-memory effect	Thermomechanical programming and recovery
	Swelling-induced morphing	Differential expansion in hydrogel systems
	Magnetothermal conversion	Remote heating triggering phase transitions
Clinical Maturity	In vitro	Bench-top or cell-based validation
	Small animal	Testing in rodent models
	Large animal	Clinically relevant anatomical models
	Human application	Early clinical or case-based use

### 3.1. Shape-Memory Materials

Shape-memory materials correspond primarily to the “shape-memory effect” mechanism defined in Table 1. These systems rely on reversible or irreversible transitions between temporary and permanent configurations induced by external stimuli, most commonly heat.

#### 3.1.1. Shape-Memory Polymers

Shape-memory polymers (SMPs) are the most widely used materials in 4D biomedical printing due to their tunable thermal transitions, relatively straightforward processing, and compatibility with extrusion-based and photopolymerization techniques [25,26]. The shape-memory effect arises from the presence of two structural components within the polymer network:

(1) a stable network that defines the permanent shape (chemical crosslinks or physical entanglements), and
(2) a switching segment that enables reversible phase transition (e.g., glass transition or crystallization).

During programming, heating above the transition temperature increases molecular mobility in the switching segments, allowing deformation. Cooling restricts chain mobility and fixes the temporary shape [27]. Upon reheating, stored elastic strain energy is released, driving recovery. From the classification perspective (Table 1), SMP-based devices are frequently categorized as irreversible systems, particularly in implantable devices such as self-expanding stents or occlusion devices, where deployment occurs once [28]. However, reversible multi-cycle SMPs have also been reported, although they are less common in clinical applications. Thermal transition temperatures are critical for biomedical use. For minimally invasive deployment, the activation temperature is typically adjusted to be slightly above body temperature to prevent premature actuation while ensuring in vivo recovery [29]. Transition temperature tuning is achieved through copolymerization, adjustment of crosslink density, incorporation of plasticizers, or blending with secondary polymers [29,30].

Common SMP materials include:

- Polylactic acid (PLA);
- Polycaprolactone (PCL);
- Polyurethane-based SMPs;
- Epoxy-acrylate networks for vat polymerization.

Biodegradable SMPs are particularly attractive for temporary implants, as gradual degradation eliminates the need for secondary surgical removal [30]. However, several challenges remain. Recovery speed may be limited in thick constructs due to thermal diffusion constraints. Mechanical stiffness often decreases near the transition temperature, potentially affecting load-bearing applications. Furthermore, achieving reliable two-way or multi-shape memory behavior increases network design complexity [31,32].

#### 3.1.2. Shape-Memory Alloys

Shape-memory alloys (SMAs), particularly nickel–titanium (NiTi), operate through solid-state phase transformations between martensite and austenite phases [33]. This crystallographic transition enables high recoverable strain and significant actuation force. Within the framework of Table 1, SMAs correspond to thermally activated shape-memory systems and may demonstrate reversible transformation when cyclically loaded. Unlike polymeric SMPs, SMAs provide superior mechanical strength and fatigue resistance, making them suitable for vascular stents and load-bearing devices. In additive manufacturing, powder bed fusion methods have been explored to fabricate SMA components [34]. However, high processing temperatures, residual stress management, and microstructural control remain technical challenges. Additionally, the integration of SMAs into polymer-dominant 4D systems requires hybrid manufacturing strategies [3]. While SMAs demonstrate higher clinical maturity than many polymer-based systems, their integration into fully printed 4D constructs remains limited.

### 3.2. Hydrogels

Hydrogels correspond to the “swelling-induced morphing” mechanism described in Table 1. These systems deform primarily through anisotropic volumetric expansion triggered by environmental stimuli such as hydration, pH variation, ionic concentration changes, or temperature shifts [35,36]. Hydrogels consist of crosslinked polymer networks capable of absorbing water without dissolving. The degree of swelling depends on polymer composition, crosslink density, and environmental conditions. Actuation arises when swelling is spatially nonuniform, generating internal stress gradients that induce bending, folding, or twisting [37].

In 4D printing, anisotropy is introduced through:

- Multimaterial layering;
- Gradient crosslinking density;
- Spatial photopolymerization control;
- Differential polymer concentration.

Hydrogel systems are often categorized as reversible systems in the taxonomy (Table 1), particularly when based on physically crosslinked networks. Their reversible swelling behavior makes them suitable for dynamic scaffolds and drug delivery devices. Hydrogels are widely investigated for tissue engineering due to their high-water content, similarity to extracellular matrix, and compatibility with cell encapsulation [37]. Extrusion-based bioprinting enables direct incorporation of living cells. However, a key limitation is the trade-off between swelling capacity and mechanical strength. High swelling ratios typically reduce stiffness, restricting load-bearing applications. Additionally, actuation kinetics are often diffusion-controlled, meaning that response speed decreases with increasing construct thickness [38,39]. Enhancing mechanical reinforcement, for example, through composite hydrogel systems, remains an active research area.

### 3.3. Liquid-Crystal Elastomers

Liquid-crystal elastomers (LCEs) combine elastomeric polymer networks with anisotropic liquid-crystal mesogen [40]. Their actuation is governed by reversible transitions between ordered (nematic) and disordered (isotropic) molecular states. Under thermal or optical activation (see Table 1, “Stimuli”), aligned mesogens lose orientation, resulting in contraction along the alignment axis [41]. During extrusion-based printing, shear forces align mesogens within the printed filament, enabling spatially programmable anisotropic deformation. In the classification framework (Table 1), LCE systems are generally reversible and capable of repeated actuation cycles. Compared with swelling-based systems, LCEs often demonstrate faster response times and greater strain magnitude (Figure 2). Potential biomedical applications include soft actuators and adaptive devices. However, challenges include precise control of mesogen alignment, material synthesis complexity, and limited long-term biological validation data [41]. 

### 3.4. Magneto-Responsive Composites

Magneto-responsive materials align with the “magnetothermal activation” mechanism described in Table 1. These systems incorporate magnetic nanoparticles, commonly iron oxide, within polymer matrices [42]. Upon exposure to alternating magnetic fields, nanoparticles generate localized heat via magnetothermal conversion. This localized heating triggers phase transitions in SMP matrices or induces mechanical deformation in elastomeric composites. These systems fall under the “magnetic stimuli” category in the taxonomy (Table 1) and are typically classified as remotely activated systems. Remote activation enables untethered deployment, which is advantageous for minimally invasive biomedical devices. Key considerations include uniform nanoparticle dispersion, prevention of agglomeration, long-term cytocompatibility, and safe magnetic field exposure levels [43]. Most reported systems remain at in vitro or small-animal validation stages (Table 1, “Clinical Maturity”).

### 3.5. Electroactive and Hybrid Systems

Electroactive materials correspond to the “electric field stimulation” category in Table 1. These systems deform under applied electric fields due to electrostatic forces or ionic migration within the material. Electroactive hydrogels can generate controllable curvature changes and enable programmable actuation. However, implantable applications are limited by power delivery requirements and potential electrochemical stability concerns. Hybrid systems integrate multiple material classes to achieve multi-stimuli responsiveness. Examples include SMP–hydrogel composites or magnetically filled hydrogels [44]. Within the taxonomy framework (Table 1), such systems combine multiple actuation mechanisms and stimulus categories. While hybrid systems enhance functionality, they increase fabrication complexity and introduce additional translational challenges [44,45]

## 4. Healthcare

The healthcare sector has shown increasing interest in 4D printing because it enables minimally invasive delivery of compact devices that can deploy on demand, as well as dynamic tissue constructs that evolve over time under physiological cues. In contrast to conventional 3D printing, where geometry and properties are essentially static, 4D printing integrates additive manufacturing with stimuli-responsive material systems (e.g., shape-memory polymers, swelling hydrogels, and responsive composites) so that printed constructs undergo programmed, predictable changes in shape or function when exposed to a defined stimulus (thermal, hydration, pH, light, electric or magnetic fields, or biological triggers) [3,15,46]. From a translational perspective, biomedical 4D systems must be designed around four coupled requirements: **(i)** a biocompatible/biodegradable material with a safe trigger window (e.g., near-body temperature, clinically acceptable magnetic fields), **(ii)** an AM method that provides sufficient resolution and repeatability, **(iii)** a mechanism that links the trigger to a controlled response (shape recovery, swelling-driven morphing, magnetothermal heating, or cell-mediated contraction), and **(iv)** clinically relevant performance metrics (deployment time, radial force, fatigue, thrombogenicity, and when degradable, mass loss and by-products) [47,48]. To support cross-study comparison and avoid purely qualitative claims, (Table 2) summarizes representative reports across stents, occluders, tissue engineering constructs, drug delivery systems and wearables, including materials, printing modality, stimulus/conditions, response, and evidence level.

### 4.1. Medical Stents

**Figure 3:**
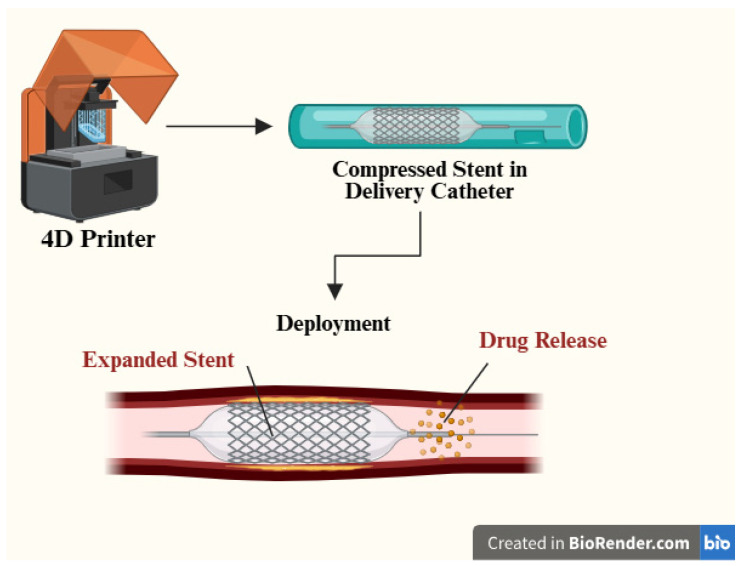
Deployable stents as an advanced application of 4D printing in biomedical systems.

Medical stents used in the trachea maintain patency in hollow anatomical structures by supporting the surrounding tissues. Traditional 3D-printed stents offer improved anatomical conformity compared with conventional devices but remain static and cannot accommodate physiological variations over time [49,50]. 4D-printed stents aim to address this by combining AM with shape-memory or swelling-driven materials so the device can be delivered in a compact configuration and then expand or reconfigure in situ under a controlled trigger [51,52]. Consequently, 4D printing is emerging as a transformative technology for developing adaptive medical stents with enhanced functionality and improved clinical outcomes (Figure 3).

#### 4.1.1. Vascular Stent

Among the most extensively studied systems are vascular stents fabricated from shape-memory polymers, including polycaprolactone (PCL), polylactic acid (PLA), polyurethane, and their composites. After printing, the stent is thermomechanically programmed into a temporary, compressed diameter; exposure to a target temperature (often selected near physiological conditions) activates the polymer’s transition (e.g., glass transition/softening), enabling shape recovery to the permanent expanded geometry and restoring lumen patency [53,54]. Zhou et al. [55] developed a bioresorbable vascular stent using methacrylated PCL fabricated via fused deposition modeling, with diameters of approximately 5 mm and lengths ranging from 10 to 40 mm. The incorporation of β-cyclodextrin enabled drug loading and sustained release, resulting in a multifunctional device that combined mechanical support with therapeutic delivery. These stents demonstrated shape recovery efficiencies approaching 100%, high tensile strength, and sustained endothelial cell proliferation, highlighting their potential for vascular regeneration and restenosis prevention. Structural design has also played a critical role in enhancing performance. Lin et al. [56] introduced auxetic stent architectures based on PLA, characterized by a negative Poisson’s ratio. These structures exhibited shape recovery efficiencies exceeding 91% and ultra-fast recovery times below 5 s, enabling rapid deployment under physiological conditions. The auxetic geometry improved radial expansion behavior and resistance to compressive forces from surrounding tissues, thereby enhancing both mechanical stability and adaptability. Such designs illustrate how geometry–material coupling can significantly influence stent performance beyond material selection alone.

#### 4.1.2. Tracheal Stent

Beyond vascular systems, 4D printing has been successfully extended to tracheal stents, which represent one of the earliest clinically translated applications of 4D-printed biomedical devices. The trachea is a critical component of the respiratory system and serves as a conduit for airflow between the larynx and the lungs. In addition to facilitating respiration, it filters, humidifies, and warms inhaled air, provides structural integrity to prevent airway collapse, and plays a role in phonation [57]. Pathological narrowing or obstruction of the tracheal lumen can lead to severe respiratory distress, which necessitates medical intervention. Tracheal stents have traditionally been used to maintain airway patency, thereby alleviating breathing difficulties and improving ventilation [20,58].

4D printing has introduced a new paradigm in tracheal stent design by enabling enhanced adaptability and reducing the risk of complications commonly associated with conventional static stents [20]. A landmark example is the polycaprolactone-based tracheobronchial splint designed for pediatric patients with tracheobronchomalacia. Fabricated using stereolithography, this device was implanted in infants and demonstrated effective airway stabilization while accommodating growth through gradual biodegradation. Clinical outcomes showed significant improvement in respiratory function, marking one of the first demonstrations of 4D printing in human patients [59]. Subsequent studies by Pandey et al. [60], and Zhang et al. [61] further advanced the application of SMPs and shape-memory polymer composites (SMPCs) in tracheal stent fabrication. These studies emphasized improved anatomical conformity and mechanical responsiveness to provide stents that better accommodate dynamic tracheal movements and patient-specific anatomical variations.

#### 4.1.3. Orbital Stent

The management of enophthalmos has been hindered by persistent clinical challenges associated with conventional implants, including suboptimal anatomical conformity, limited volumetric filling efficiency, invasiveness of surgical placement, and lack of radiographic visibility for post-operative assessment. 4D-printed orbital stents have been developed to restore anatomical structure following trauma or tumor resection. Deng et al. [62] fabricated shape-memory polyurethane-based stents incorporating gold nanoparticles and nanohydroxyapatite. These composite systems enabled minimally invasive insertion in a flattened state, followed by thermally induced shape recovery to restore orbital volume. The inclusion of gold nanoparticles provided enhanced imaging visibility, while nanohydroxyapatite promoted osteointegration. These devices demonstrated excellent biocompatibility, structural stability, and functional restoration, highlighting the potential of 4D printing in craniofacial reconstruction.

#### 4.1.4. Intestinal Stents

Intestinal stents are devices designed to alleviate obstructions within the gastrointestinal tract. The integration of 4D printing technology into stent fabrication offers advantages in terms of customizability, flexibility, and responsiveness to physiological conditions. However, developing intelligent stents presents a challenge for achieving appropriate transition temperatures for safe implantation. The trigger temperature must be safe for surrounding tissue and compatible with deployment in a wet, compliant environment. To address this gap, Lin et al. [63] reported PEG/PLA-based stents capable of shape recovery at near-body temperature in hydrated environments. These devices exhibited stable expansion under physiological conditions and effective obstruction relief in swine models, demonstrating their potential for translational use. Importantly, the activation temperature was carefully tuned to align with physiological conditions, ensuring safe deployment without thermal damage to surrounding tissues.

Across these applications, several key performance metrics have been consistently reported. Shape-memory stents typically exhibit recovery ratios above 90%, response times ranging from seconds to minutes depending on activation mechanism, and sufficient mechanical strength to withstand physiological loading conditions. However, the specific performance is strongly influenced by both material composition and structural design, emphasizing the importance of integrated system optimization.

Despite significant progress, several challenges remain before widespread clinical adoption can be achieved. Precise control of activation conditions is critical, particularly for thermally triggered systems, where excessive heat may damage surrounding tissues. Mechanical durability under long-term cyclic loading, especially in vascular environments, remains an important concern. In addition, achieving predictable degradation behavior for biodegradable stents is essential to ensure that structural support is maintained during the healing process while avoiding long-term complications. Overall, 4D-printed stents demonstrate the highest level of translational maturity among 4D biomedical applications, with successful progression from in vitro studies to large-animal models and early clinical use. Their ability to integrate programmable deployment, multifunctionality, and patient-specific design positions them as a key driver for the clinical translation of 4D printing technologies.

### 4.2. Cardiac Occluders

Cardiac occluders represent a critical application of 4D printing in cardiovascular medicine, where devices are required to achieve reliable sealing of structural defects such as atrial septal defects (ASD) and left atrial appendage (LAA) openings. Conventional occluders fabricated from nitinol alloys provide high mechanical strength but are associated with long-term complications, including metal ion release, chronic inflammation, and limited adaptability to patient-specific anatomy [63,64]. In this context, 4D printing offers a transformative approach by enabling biodegradable, stimuli-responsive occlusion devices with programmable deployment behavior.

**Figure 4:**
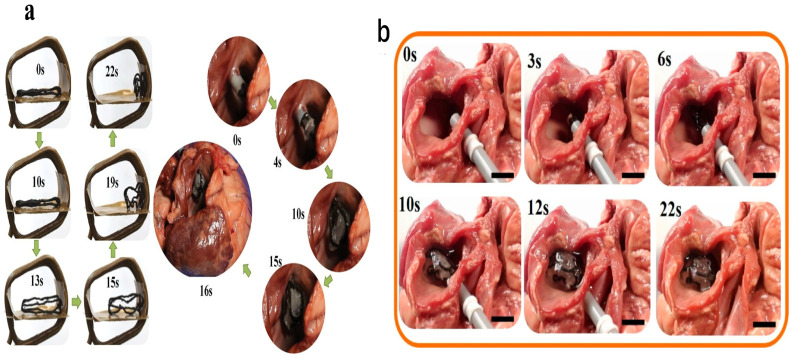
(**a**) Magnetically actuated shape recovery behavior of a programmed polylactic acid (PLA) occluder (**left**), along with an in vitro evaluation demonstrating its functional applicability (**right**). Reprinted with permission from Ref [65] copyright 2019 John Wiley and Sons. (**b**) Experimental validation of transcatheter occlusion of the left atrial appendage, highlighting the device’s feasibility. Scale bars represent 10 mm. Reprinted with permission from Ref [66]. © 2021 American Chemical Society.

Recent studies have demonstrated the feasibility of magnetically responsive polymer composites for occluder applications. Lin et al. [65] developed 4D-printed occluders based on polylactic acid (PLA) integrated with Fe_3_O_4_ nanoparticles. These devices were fabricated using fused deposition modeling and programmed into compact configurations suitable for catheter-based delivery. Upon exposure to an alternating magnetic field, the embedded magnetic nanoparticles generated localized heat via magnetothermal conversion, triggering shape recovery of the polymer matrix. The resulting deployment was rapid, typically occurring within seconds, with shape recovery efficiencies approaching 95% and stable defect sealing performance. This remote actuation mechanism offers several advantages over thermally triggered systems, including non-contact activation and improved spatial control, which are particularly important for delicate cardiovascular environments. In addition, in vivo studies demonstrated that these devices maintained structural integrity and achieved effective occlusion without significant thrombus formation or inflammatory response. Histological analyses further confirmed the absence of pathological changes in major organs, indicating favorable biocompatibility (Figure 4a). Building on this concept, Lin et al. [66] developed bioinspired LAA occluders using similar magneto-responsive systems, incorporating structural designs that mimic natural anatomical features. These devices demonstrated enhanced conformability to irregular geometries and improved sealing efficiency (Figure 4b). Moreover, the use of biodegradable polymers allows gradual resorption of the device over time, reducing long-term complications associated with permanent implants [66]. In parallel, digital light processing has been used to fabricate patient-specific occluders with tunable mechanical properties and transition temperatures. These systems enable fine control over stiffness and deployment behavior, allowing better matching with soft cardiac tissues. In vitro studies have shown high cell viability and minimal cytotoxicity, while preliminary in vivo evaluations suggest promising performance [16]. Despite these advances, several challenges remain. Uniform heating during magnetothermal activation must be carefully controlled to avoid localized overheating, and long-term degradation behavior must be optimized to ensure predictable device performance. Additionally, achieving regulatory approval for such multifunctional devices will require extensive validation of safety and reliability. Overall, 4D-printed cardiac occluders demonstrate strong translational potential, combining remote actuation, biodegradability, and patient-specific design, positioning them as promising alternatives to conventional metallic devices.

### 4.3. Tissue Engineering

Tissue engineering aims to restore damaged tissues by generating functional surrogates [67]. Despite notable advances, most protocols still culture cells within static scaffolds, limiting anatomical fidelity and biomechanical stimulus. Morphodynamic matrices rectify this shortcoming: their programmed shape change yields construct that fit defect sites precisely while providing a dynamic milieu that augments cell-cell and cell-matrix interactions, thereby accelerating organisation, remodelling and maturation [68]. Controlled transformations from simple precursors to complex architectures further enable intricate designs beyond the reach of conventional methods. 4D printing seeks to overcome this by using shape-morphing matrices that undergo controlled transformations under defined cues, enabling constructs to better conform to defect geometries and provide a dynamic microenvironment that can influence cell organisation and maturation.

#### 4.3.1. Vascular Tissue Engineering

**Figure 5:**
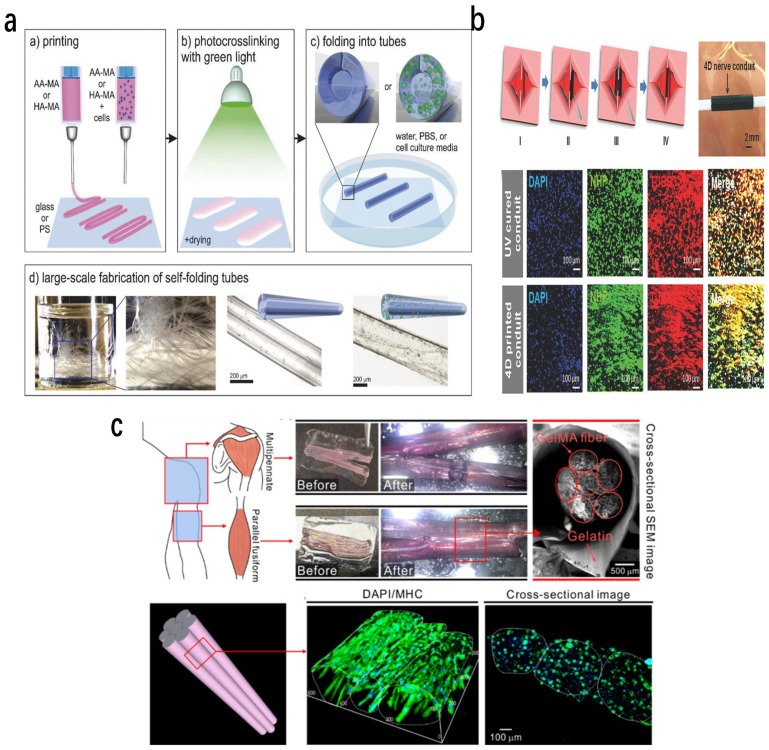
(**a**) Schematic overview of the 4D biofabrication workflow for constructing cell-laden hydrogel tubes, including the sequential steps of bioprinting, photo-crosslinking, and shape transformation of AA-MA or HA-MA hydrogels (**top**), along with representative examples of the resulting self-folded tubular structures (**bottom**). Reprinted with permission from Ref [71]. copyright 2017 John Wiley and Sons (**b**) Stepwise illustration of the entubulation process using a 4D reprogrammable nanohybrid nerve guidance conduit based on thermally induced shape recovery. (I) Representation of a transected peripheral nerve with two stumps. (II) Placement of the shape-programmed flat nanohybrid conduit beneath the injury site. (III) Reversion of the conduit to its original tubular configuration upon exposure to physiological temperature. (IV) Complete encasement of the nerve stumps by the recovered conduit. The upper image shows surgical integration of the 4D-printed device, while the lower panel presents immunofluorescence staining of neurogenically differentiated human mesenchymal stem cells (hMSCs) on both 4D-printed and UV-cured conduits. Reprinted with permission from Ref [74]. copyright 2018 John Wiley and Sons. (**c**) Fabrication of bundled, cell-laden GelMA fibers via folding of a gelatin-based film (**top**), accompanied by three-dimensional microscopy images showing the structural integrity and cell distribution in the bundled GelMA fibers following 21 days of culture. Reprinted from Ref [77].

The vasculature plays a key role in sustaining native tissues, including bone, muscle, and skin, by delivering oxygen and nutrients while removing metabolic waste. In tissue engineering, diffusion-dependent nutrient transport suffices for small constructs (<200 μm); however, it cannot meet metabolic demands of larger engineered tissues, resulting in hypoxia, apoptosis, and structural failure [69]. Recapitulating functional vascular networks remains an imperative, unresolved challenge, limiting clinical translation of engineered tissues. Traditional fabrication methods struggle to replicate the hierarchical architecture of blood vessels, particularly in achieving microscale luminal diameters without compromising structural resolution [70]. Emerging 4D printing strategies using dynamic shape-morphing materials offer a promising avenue for generating tunable vascular-like structures. Kirillova et al. [71] developed shape-morphing hydrogels based on methacrylated alginate and hyaluronic acid. Using photopolymerization techniques, ultrathin films were fabricated and subsequently transformed into tubular structures through anisotropic swelling. These constructs achieved lumen diameters ranging from 20 to 150 μm, closely matching the scale of microvascular networks, while maintaining high cell viability (Figure 5a). However, challenges remain in scaling these systems to replicate hierarchical vascular architectures and ensuring long-term perfusion [71].

Despite advances, current 4D printing techniques remain confined to generating microtubes of set dimensions, failing to replicate native vasculature’s anatomical complexity and multilayered architecture. Challenges persist in ensuring wall fusion post-folding to prevent leakage and validate long-term maturation of printed networks. Future research must prioritize developing multiscale multilayered vascular constructs and strategies to enhance endothelialization and integration. These improvements will advance the clinical relevance of 4D-bioprinted vasculature in regenerative medicine.

#### 4.3.2. Neural Tissue Engineering

Peripheral nerve injuries with critical gaps (>1 cm) from severe trauma require clinical intervention [72]. Artificial nerve guidance conduits (NGCs) are the gold standard for bridging nerve stumps; however, conventional fabrication methods like injection molding and 3D printing face limitations from labor-intensive protocols, structural fragility, and handling risks [73]. These constraints necessitate innovative NGC fabrication approaches. For nerve guidance conduits, 4D strategies include both stress-programmed self-folding sheets and swelling-programmed self-rolling hydrogels. Miao et al. [74] fabricated graphene-enhanced shape-memory polymer conduits that undergo self-folding into tubular structures, providing guidance for axonal growth. These systems demonstrated enhanced neural differentiation and alignment of stem cells, as well as improved regeneration outcomes in small-animal models (Figure 5b). Addressing this gap, Joshi et al. [75] engineered an alginate-methylcellulose hydrogel system with tunable swelling kinetics for 4D bioprinting of self-rolling NGCs. Implanted into a 2 mm sciatic nerve defect model in Sprague-Dawley rats, the conduits achieved suture-free nerve coaptation. Histomorphometric and electrophysiological evaluations at 6 weeks post-implantation confirmed axonal regrowth and functional recovery, validating 4D-printed NGCs in peripheral nerve repair.

#### 4.3.3. Muscle Tissue Engineering

Skeletal muscle derives its contractile strength from a hierarchical architecture in which aligned myofibers aggregate into fascicles wrapped by epimysium [76]. Traumatic defects disrupt this structure and can impair motor function or precipitate organ failure. Conventional engineering strategies align myoblasts on 2D substrates but have difficulty recreating the 3D hierarchy required for physiological force generation [76]. 4D printing provides a route for bridging this gap. Using a bespoke extrusion platform, Yang et al. [77] printed C2C12-laden hydrogel filaments while applying an electric field to orient cells (Figure 5c). The fibers were deposited onto a grooved gelatin sheet fabricated by 4D printing; differential swelling drove the sheet to self-roll, bundling the filaments into fascicle-like units that recapitulated the native muscle organization. The construct supported robust myotube formation, demonstrating that programmable morphodynamics can convert planar alignment cues into functional three-dimensional muscle tissue.

Cardiac muscle presents a complementary challenge, the ventricular wall is intrinsically curved, which complicates the seamless integration of planar grafts. Engineered surface topologies improve myocardial repair [78], yet matching the curvature of the heart remains elusive. Wang et al. [79] developed 4D-printed cardiac patches using polyethylene glycol diacrylate (PEGDA) molds combined with graphene-enhanced shape-memory polymer composites. These constructs were initially fabricated as flat sheets to facilitate cell seeding and alignment through microgroove structures. Upon near-infrared (NIR) stimulation, the patches transformed into curved geometries matching the ventricular surface. This approach enabled uniform cell distribution, enhanced myocardial alignment, and improved tissue integration, demonstrating the potential of 4D printing for functional cardiac repair.

#### 4.3.4. Bone Tissue Engineering

**Figure 6:**
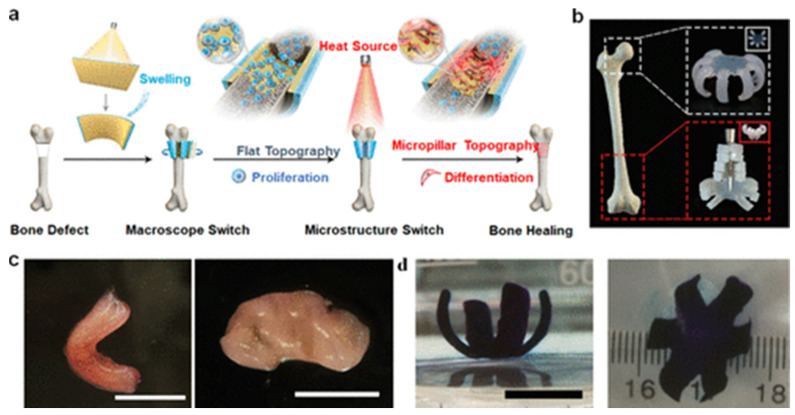
(**a**) Schematic representation of a 4D printed bilayer membrane integrating a shape memory polymer (SMP) and hydrogel, designed for applications in bone tissue regeneration. (**b**) Customization of the bilayer membranes to conform to patient-specific anatomical geometries. (**a**,**b**) reprinted with permission from Ref [81]. copyright 2021 John Wiley and Sons. (**c**) Illustrative examples of 4D-fabricated cartilage tissues engineered into complex geometries, including a “C”-shaped construct (**left**) and a spiral structure (**right**). Scale bar: 10 mm. Reprinted from Ref [85]. (**d**) Bioengineered cartilage tissues exhibiting floral morphologies, with designs comprising four petals (**left**) and six petals (**right**). Scale bar: 1 cm. Reprinted with permission from Ref [86]. copyright 2022 John Wiley and Sons.

Bone tissue engineering has emerged as a critical discipline for addressing bone tissue repair compromised by congenital anomalies, trauma, or degenerative pathologies [80]. Conventional strategies using static scaffolds are constrained by their inability to adapt to complex bone defect geometries, often requiring invasive surgery. By contrast, 4D printing, integrating dynamic, stimuli-responsive materials, enables fabrication of patient-specific constructs capable of morphological adaptation in situ, minimizing surgical invasiveness [75]. You et al. [81] pioneered this approach by engineering bilayer 4D-printed constructs comprising a shape-memory polymer (SMP) layer and a hydrogel actuator. The SMP layer, fabricated via thermal curing in polydimethylsiloxane (PDMS) molds, exhibited reversible switching between flat and micropillar topographies, while the hydrogel layer, photocured using digital light processing (DLP), drove shape adaptation via swelling. This system achieved defect-specific conformation for minimally invasive implantation while regulating osteogenic cell behaviors through tunable surface topographies (Figure 6a,b). These constructs achieved shape recovery efficiencies of approximately 80–90%, although activation temperatures around 46 °C remain higher than ideal physiological conditions.

Complementary innovations include Wang et al. [82]’s direct ink writing (DIW) of β-tricalcium phosphate (β-TCP)/shape-memory poly(lactic acid-co-trimethylene carbonate) scaffolds with black phosphorus nanosheets. These constructs underwent photothermally triggered shape recovery under near-infrared (NIR) irradiation, enabling precise defect filling in cranial bone models. Similarly, Zhou et al. [83] developed magnetoresponsive PLA/Fe_3_O_4_ SMP scaffolds that restored architecture via magnetic-field-induced magnetothermal effects. Both strategies demonstrate 4D printing’s potential in combining anatomical precision with reduced surgical trauma. This demonstrates the synergy of 4D printing with bioactive stimuli to augment regenerative efficacy. Despite its promise in bone tissue engineering, 4D printing still faces challenges in tuning activation temperatures, ensuring biocompatibility, and mimicking bone’s mechanical gradients. Future efforts should prioritize in vivo validation and vascular integration to enable volumetric regeneration. With dynamic materials and clinical scalability, 4D printing offers a new paradigm for precise bone repair.

#### 4.3.5. Cartilage Tissue Engineering

Cartilage possesses limited intrinsic self-healing capacity, often necessitating surgical intervention. Despite advances, achieving full functional restoration and recapitulation of native tissue architecture remains a formidable clinical challenge. Cartilage regeneration represents a critical focus in orthopedics, driven by the urgent need to address injuries and pathologies such as osteoarthritis [84]. Conventional static scaffolds, akin to those used in bone tissue engineering, face inherent limitations in replicating the dynamic biomechanical and structural complexity of native cartilage. 4D printing technologies, however, offer transformative potential for generating patient-specific solutions through spatiotemporal control of scaffold behavior.

Recent advancements have leveraged biosourced materials, including gelatin and alginate derivatives, to engineer 4D-printed cartilage constructs [85,86]. Ding et al. [86] synthesized gelatin methacryloyl (GelMA) and oxidized methacrylated alginate (OMA) hydrogels with tunable swelling properties were synthesized and processed into cell-laden bioinks via mechanical jamming. These constructs demonstrated high-resolution printability and underwent programmed or stimulus-responsive morphological reconfiguration upon hydration in culture media. When seeded with human mesenchymal stem cells (hMSCs) and cultured under chondrogenic conditions, the constructs matured into complex tissues rich in cartilage-specific extracellular matrix components, including glycosaminoglycans and collagens (Figure 6c,d). Despite these promising developments, the translation of 4D-printed tissue engineering systems is limited by challenges including insufficient mechanical strength, lack of vascularization, and difficulty in scaling complex constructs. Nevertheless, the ability to integrate structural adaptability with biological functionality positions 4D printing as a powerful platform for regenerative medicine.

### 4.4. Drug Delivery Systems (DDSs)

Nanoscale carriers enhance bioavailability by localizing therapeutics to target sites and enabling controlled release. However, their clinical effectiveness is often compromised by the complex physiology of the body, which can provoke nonspecific retention or premature payload leakage during circulation [87]. 4D printing can enhance drug delivery by coupling geometry change to triggered or delayed release, enabling devices that deploy after administration (e.g., gastroretentive or intravesical systems) and then sustain local therapy. Because clinical translation requires tight control of dose, release kinetics, and device integrity, the most convincing examples explicitly link stimulus → morphing → release mechanism [88,89].

Wang et al. [90] employed drug-loaded hydrogels printed with shape-fixing chemistries to demonstrate proof-of-concept release modulation, but neither in vivo performance nor the mechanistic link between morphing and release kinetics was thoroughly examined. To solve this problem, subsequent studies have begun to integrate shape changes with pharmacokinetics. Helical SMP constructs, thermomechanically programmed and encased in gelatin capsules, were shown to self-expand under physiological conditions, triggering sustained release only after deployment [91]. Intravesical therapy is a promising treatment option. Using hot-melt extrusion and fused-deposition modelling, PVA-based shape-memory polymers were printed into devices that could be inserted in a compact form, recover their original geometry upon hydration, and gradually erode to release encapsulated drugs [91]. Systematic evaluation confirmed the water-driven shape recovery, controllable swelling, mass loss, and prolonged drug elution, underscoring the suitability of PVA SMPs for bladder-resident DDSs.

Hydrogel-based systems provide additional control through microstructural modulation. Poly(N-isopropylacrylamide) (PNIPAM)-based constructs exhibit temperature-responsive behavior, enabling reversible opening and closing of pores. This allows dynamic regulation of drug diffusion, providing a dual mechanism of control combining macroscopic deformation and microscopic transport modulation [92,93]. These examples illustrate how device-scale morphing (unfolding/expansion) and microstructure-scale changes (pore gating) can both be harnessed for programmable delivery. Despite these advantages, significant challenges remain. The relationship between structural transformation and pharmacokinetics is not yet fully understood, and achieving reproducible dosing remains difficult. In addition, regulatory pathways for such combination products are complex, requiring rigorous evaluation of both device and drug components.

### 4.5. Wearables

Wearable technologies are transforming healthcare by enabling continuous, non-invasive monitoring and personalized assistance [94]. 4D printing extends this paradigm by furnishing devices that dynamically conform to individual anatomy, thereby improving comfort, mobility and durability [95]. Polymers and composites dominate this field because they combine low density, high flexibility, and mechanical resilience.

**Figure 7:**
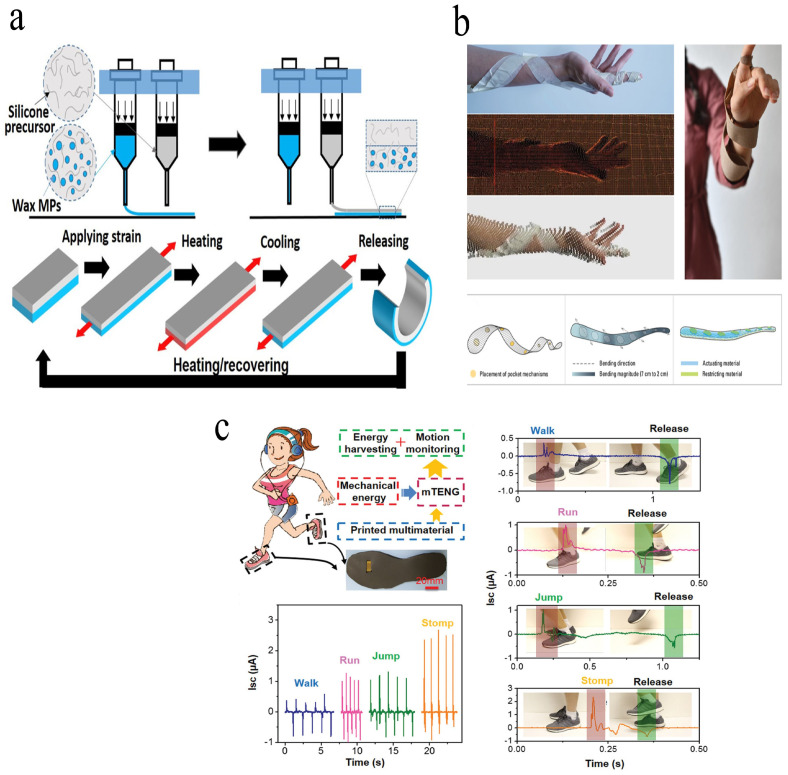
(**a**) Schematic overview of the 4D printing process used to fabricate a bilayer silicone composite film, along with its application in electrocardiogram (ECG) wearable devices for real-time heartbeat monitoring. Reprinted with permission from Ref [97]. copyright 2021 American Chemical Society. (**b**) Design and fabrication of a self-adjusting wrist–forearm splint through a top-down inverse modeling strategy , and its practical validation demonstrated by user wearability. Reprinted from Ref [98]. (**c**) Application of a micro-triboelectric nanogenerator (mTENG) integrated into a shoe insole for dynamic motion tracking. Reprinted with permission from Ref [99]. copyright 2019 John Wiley and Sons.

Thermally responsive shape-memory materials, notably poly (lactic acid) (PLA), allow the printed construct to switch from a rigid to a compliant state after precise heating. This capability has been exploited to produce custom prosthetic sockets that self-adjust to residual-limb geometry, reduce fit-related discomfort, and streamline the fitting process [96]. More recently, multimaterial 4D printing has been harnessed to integrate electronic functions directly into morphing substrates. Deng et al. [97] used a dual-nozzle printer to build shape-adaptive electrocardiography (ECG) patches whose curvature matches the thorax, yielding high-fidelity cardiac signals (Figure 7a). Beyond sensing, biomimetic strategies now target active assistance: Cheng et al. [98] replicated plant-twisting mechanics to create hygroscopically driven splints that self-tighten around a limb, offering adaptive support with a modular, self-shaping architecture (Figure 7b). Similarly, Cheng et al. [99] fabricated triboelectric nanogenerators (TENGs) capable of hierarchical shape change, embedded in shoe insoles, and harvested mechanical energy while tracking the gait (Figure 7c). Transparent, self-recovering TENGs printed from shape-memory polymers coated with silver nanowire electrodes further demonstrated the real-time sensing of joint flexion [100].

Despite these advances, most 4D-printed wearables remain confined to proof of concept demonstrations that focus on monitoring rather than therapy. Translational progress will require deeper integration of actuation, power, and data communication along with rigorous evaluation under clinical conditions. Nevertheless, ongoing improvements in printing resolution, multi-material processing, and stimuli-responsive chemistries signal a fertile future, in which 4D wearables deliver patient-specific, non-invasive interventions across rehabilitation, drug delivery, and soft robotic assistance.

**Table 2:** Comparative Overview of 4D-Printed Biomedical Applications.

Application	AM Technique	Material System	Stimulus & Activation Conditions	Mechanism of Response	Functional Outcome/Clinical Relevance	Evidence Level	References
Vascular Stents	FDM	PCL; PLA; βCD-g-PCL; PLA/Fe_3_O_4_ composites	Thermal activation (~37–45 °C); AMF-induced magnetothermal heating	Thermomechanical programming → shape recovery; magnetothermal-triggered expansion	Catheter delivery; in situ radial expansion; potential drug release	In vitro; small animal	[53,54,55,101]
Tracheal/Airway Stents	SLA; DLP; FDM	PCL; SMP composites	Body-temperature activation (~37 °C)	Shape-memory expansion; controlled biodegradation	Airway patency; pediatric growth accommodation	Small animal; early human use	[59,60,61]
Intestinal Stents	FDM	PEG/PLA near-body-temperature biocomposites	Thermal activation at physiological temperature	Shape recovery in hydrated conditions	Relief of obstruction in swine model	Large animal	[63]
Cardiac Occluders (ASD/LAA/VSD)	FDM; DLP	PLA/Fe_3_O_4_ composites; magnetic PLA; bioresorbable elastomers	Alternating magnetic field (AMF); thermal activation	Magnetothermal heating → SMP transition → rapid recovery	Transcatheter defect closure; biodegradable alternative to nitinol	In vitro; large animal (long-term)	[16,65,66]
Vascular Tissue Engineering Constructs	DIW + photopolymerization	Methacrylated alginate (AA-MA); methacrylated HA (HA-MA)	Hydration/culture media exposure	Anisotropic swelling → self-rolling microtubes	Microvascular-like structures (20–150 μm lumen); maintained viability	In vitro	[71]
Neural Guidance Conduits	SLA; extrusion bioprinting	SOEA/graphene composites; alginate–methylcellulose hydrogels	Solvent exposure; hydration; physiological temperature	Stress-gradient folding; swelling-driven self-rolling	Suture-free nerve repair; axonal regeneration	Small animal (rat)	[74,75]
Bone Scaffolds	DLP; DIW	SMP–hydrogel bilayers; β-TCP/SMP composites; PLA/Fe_3_O_4_ systems	Thermal (~46 °C); NIR photothermal; magnetic stimulation	Shape recovery; photothermal/magnetothermal activation	Defect-specific conformation; osteogenic enhancement	In vitro; small animal	[81,82,83,86]
Cartilage Constructs	Extrusion bioprinting	GelMA; oxidized methacrylated alginate (OMA) hydrogels	Hydration-induced swelling	Differential swelling → programmed geometric transformation	Complex cartilage geometries; ECM deposition	In vitro	[86]
Drug Delivery Systems	FDM; hot-melt extrusion; extrusion	PVA-based SMPs; PNIPAM hydrogels	Hydration; physiological temperature; thermoresponsive phase transition	Shape expansion; pore opening/closing	Prolonged local drug release; gastric retention	In vitro; limited in vivo	[91,92,93]
Wearable Biomedical Devices	Multi-material extrusion; dual-nozzle printing	PLA SMPs; elastomeric composites; conductive composites	Thermal activation; humidity; mechanical deformation	Shape adaptation; stiffness modulation	Conformal fitting; sensing and energy harvesting	Prototype; human volunteer testing	[96,97,98,99]

## 5. Concluding Remarks, Challenges and Future Prospectives

Advancements in 4D printing have driven significant progress in healthcare, particularly in tissue engineering, biomedical devices, and drug delivery systems. Additionally, it imparts functional benefits, including self-healing, improved controllability under stimuli, and support for precision-targeted therapy. This emerging technology enables the creation of highly customized, user-centric solutions, setting the stage for transformative applications. Ongoing research focuses on discovering novel materials, expanding design complexity, and refining printing techniques developments that promise to further enhance the field. Together, these innovations are poised to improve human health and well-being.

Current efforts focus on expanding materials, increasing complexity, and refining fabrication processes to improve patient care. In 4D bioprinting of biomedical constructs, bioinks must enable scalable production and high-resolution printing under physiological conditions while maintaining cell viability. Cells should not hinder shape-morphing behaviors and must retain functional responses.

### 5.1. Translational Considerations and Clinical Challenges

Although 4D printing shows strong potential for biomedical implants and functional healthcare devices, several translational barriers must be addressed before clinical implementation.

Sterilization compatibility is a primary concern. Many stimuli-responsive polymers and hydrogels are sensitive to conventional sterilization methods. Autoclaving may deform thermally responsive shape-memory polymers, while gamma irradiation and electron-beam exposure can alter polymer chain integrity and mechanical performance. Ethylene oxide sterilization is often more suitable for temperature-sensitive materials, but residual removal must be carefully validated. Material-specific sterilization protocols are therefore required to preserve actuation properties and biocompatibility.

Manufacturing reproducibility is equally critical. Clinical translation requires consistent actuation performance across batches. Variations in dimensional accuracy during multi-material printing, differences in polymer composition, and fluctuations in shape-fixity and recovery ratios may compromise reliability. Fatigue resistance under cyclic physiological loading must also be verified. Standardized quality control procedures, including mechanical testing, thermal analysis, and actuation repeatability assessment, are essential for regulatory approval.

The safety of activation stimuli must be strictly controlled. Thermal activation should remain below cytotoxic temperature thresholds, particularly during prolonged exposure. Magnetothermal systems must avoid unintended tissue heating, and near-infrared stimulation may be limited by tissue penetration depth. Clinical use therefore requires precise stimulus modulation and controlled activation.

Biocompatibility and degradation behavior are central for implantable systems. Degradation kinetics must align with tissue healing while avoiding toxic by-products. Inflammatory response, long-term integration, and thrombogenicity in vascular applications must be evaluated. Magnetic resonance imaging compatibility should also be considered for magnetically responsive devices. Comprehensive in vivo validation remains necessary.

Regulatory pathways for stimuli-responsive implants may involve additional requirements compared to passive devices, including durability testing, accelerated aging studies, degradation assessment, and verification of actuation reliability under physiological conditions. Clear regulatory classification will influence approval strategies and development timelines.

### 5.2. Future Perspectives

Future research should focus on modulating cell behavior and using cell-generated forces to direct shape transformations. 4D-bioprinted constructs may benefit from mimicking tissue development, requiring systems that coordinate shape changes with biological timescales. Future generations of multifunctional printheads could be designed to include the fourth dimension during the printing process, allowing for a truly 4D printing experience.

Advanced Manufacturing Strategies

While 4D printing focuses on time-dependent transformation enabled by stimuli-responsive materials, further refinement of manufacturing strategies may improve structural precision and mechanical reliability. Multi-axis additive manufacturing, sometimes described as “5D printing,” refers to printing along curved toolpaths using five-axis motion control rather than strictly planar layer-by-layer deposition. This approach can enhance interlayer bonding and mechanical anisotropy control in load-bearing structures. In the context of biomedical implants, such strategies may improve mechanical integrity and durability, particularly for bone substitutes and structural components. However, 5D printing does not inherently introduce time-dependent actuation and should be considered a complementary manufacturing refinement rather than an extension of 4D functionality. Further experimental validation is required to determine its clinical relevance in biomedical applications.

Integration of Artificial Intelligence and Machine Learning

The design of 4D-printed systems involves complex relationships among material composition, printing parameters, programmed geometry, and stimulus-response behavior. Artificial intelligence (AI) and machine learning (ML) tools may assist in optimizing these multivariable systems. Potential applications include:

- Predicting shape-recovery behavior based on polymer composition and thermal properties;
- Inverse design of structures to achieve predefined morphing pathways;
- Optimization of multi-material interfaces;
- Quality control through defect detection and actuation performance monitoring.

Data-driven approaches may reduce experimental iteration and accelerate material screening. Nevertheless, reliable implementation requires standardized datasets, reproducible mechanical characterization, and validation across different printing platforms. AI-based design tools must remain grounded in experimentally verified material models to ensure safe clinical translation.

Biological Integration and Long-Term Evaluation

Beyond fabrication advances, future research must address biological complexity. For tissue-engineered constructs, integration with host vasculature, immune modulation, and long-term remodeling remain central challenges. For implantable devices, durability under cyclic physiological loading and compatibility with imaging modalities require further evaluation. Controlled coordination between material transformation and biological healing timelines is particularly important for degradable systems.

## Data Availability

No new data were created or analyzed in this study. Data sharing is not applicable to this article.

## Abbreviations

FDM	Fused Deposition Modeling
SLA	Stereolithography
DLP	Digital Light Processing
DIW	Direct Ink Writing
SMP	Shape Memory Polymer
PCL	Polycaprolactone
PLA	Polylactic Acid
PEG	Polyethylene Glycol
PVA	Polyvinyl Alcohol
PNIPAM	Poly(N-isopropylacrylamide)
AMF	Alternating Magnetic Field
HA	Hyaluronic Acid
ECM	Extracellular Matrix
